# Progress in Psychiatry

**Published:** 1899-10-21

**Authors:** 


### PROGRESS IN PSYCHIATRY.

### A SKETCH OF THE PAST.

**The Old Era.**—The growth and progress of psychiatric medicine illustrates clearly the absolute dependence of practical progress upon the acquisition and growth of scientific knowledge. In early ages the insane were looked upon as possessed by spirits good or evil, and the records of ancient Egypt, Babylon, and Judea are full of such accounts. Biblical examples of madness are too numerous to mention. The insanity of Saul, into whom "an evil spirit" had entered, of Nebuchadnezzar, who wandered in the fields "and did eat grass as oxen," and neglected his person "till his hair was grown like eagles' feathers, and his nails like birds' claws," and likewise the feigned insanity of David are household knowledge.

Even the cultured and artistic Greek was not free from such beliefs. The Hellenic mind regarded insanity as a visitation of the gods. Homer tells us how the anger of the gods was visited on Bellerophon, who became a melancholiac; Sophocles describes the furious and destructive mania of Ajax, which drove him to defy Olympus and the thunderbolts of Jove; Euripides gives a vivid description of madness sent by Juno, attacking Hercules and throwing him into an epileptic fit, which roused in him a violent homicidal phrenzy in which he destroyed his own children.

Hippocrates, the father of medicine, takes, however, a rational, and, as must even be conceded by modern physicians, a scientific view of mental disease. Thus, in regard to epilepsy—the "sacred disease" as it was then called—he observes: "The sacred disease is in no wise more Divine or sacred than other diseases, but has a natural cause, from which it originates like other affections. . . . . They who first referred this disease to the gods appear to me to have been such persons as the conjurors, purificators, mountebanks, and charlatans now are, . . . persons using the Divinity as a pretext and screen for their own inability to afford any assistance . . . and for which they have instituted a mode of treatment which is safe for themselves, viz., by applying purifications and incantations, and enforcing abstinence from baths and many articles of food which are unwholesome to men in disease. . . . This disease is formed from those things which enter into and go out of the body, and it is not more difficult to understand and cure than the others, neither is it more divine than other diseases. . . . Men ought to know that from nothing else but the brain come joy, despondency, and lamentation . . . and by the same organ we become mad and delirious." Pithy, pertinent, and sensible observations these, and it is a pity they remained but little heeded for two thousand years!

Hippocrates also proposed treating the insane by purging and ridding the body of brain-disturbing "humours"—in modern language we would say "toxic agents." His teachings and precepts, however, remained neglected for many centuries, the only exception being found in the doctrines and practice of Cælius Aurelianus in the first century A.D., who taught and practised gentleness, humane care, and an enlightened treatment of lunatics, protesting in the strongest manner against chains, fetters, and the cell, with which lunatics were at that time universally treated. After his death came an age of ignorance and barbarism. Lunatics were shut up in jails and prisons, or treated with sticks and stones if found wandering at large. The ravings of the maniac were construed as blasphemies, and the stake was often his lot. Women thus afflicted or possessed of delusions were burnt as witches and agents of the Evil One. There was little improvement in treatment for 1,600 years.

**The New Era.**—A new era arose with Pinel, the great apostle of reform and of humane treatment, who flourished at the close of the eighteenth century. Pinel was physician to the Bicêtre, the great French prison for the insane in Paris. He had what his contemporaries did not possess, an elementary but scientific knowledge of mental disease. He was also a man of courage and energy, with strong convictions of right and wrong, and with a clear conception of his duties. His reforms began at the Bicêtre, and in spite of obloquy, ridicule, and opposition, and even personal danger, he persevered in carrying out his task. In his day the picture of the chained and imprisoned lunatics was gruesome and horrible. They lived in suffering, in chains, and in filth. In less than a week he had freed more than 50 of these unfortunates from their manacles. Provision had been made beforehand by him for their proper supervision, care, and treatment, and his success was assured. Patients were found who had been in chains for 10, 20, and even 30 years. Dr. Pargeter, a contemporary of Pinel, says that the wounds and mortification caused by the chains and fetters had in some cases actually destroyed the flesh of the extremities. The horrors thus disclosed were gradually abolished. Wherever Pinel went he brought light and air, decent food and clothing, gentle and humane treatment. The days of chains and cords, and of brutal gaolers with their sticks and dogs, were numbered, and Pinel lived to see his reforms gradually crowned with success. It was Pinel's good fortune that he found a staunch and worthy successor in Esquirol. For 25 years this great disciple of an illustrious master laboured in carrying out the same humane reforms. The wave of philanthropy spread into other countries. William Tuke at the York Retreat in England, Chiarugi at the Florentine Asylum in Italy, Guislain in Belgium, and Jacobi in Germany, all laboured earnestly and diligently as pioneers in the great movement. The period of humanitarian treatment had begun.

The early half of the nineteenth century witnessed the steady and gradual growth of the new doctrines and principles. It was mainly an epoch of transition, of inquiry, and of unrest, such as attends the early years of any great reformation. The second half of the nineteenth century bore its fruit; the harvest was gathered in from the seed sown in the earlier decades. A more active and general interest was roused in the public mind concerning the welfare of the insane. Legislation was inaugurated and public asylums and hospitals opened for the care and treatment of lunatics. In this epoch, which may be designated the period of State control and of humane medical treatment, the welfare of the insane, as regard housing, comfort, and general surroundings, reached a high degree of excellence.

In the closing decade of our century we are standing on the threshold of a new epoch, in which insanities are beginning to be studied and investigated as other diseases; progress, steadily but surely, is making its way in the growth of knowledge regarding ætiology, hereditary conditions, pathology, and therapeutics; the brain, as the affected organ, and every other part of the body as partners in the morbid manifestations which in their totality we call insanity, are receiving examination with all the wealth of methods and means that modern science supplies; statistical and sociological studies are contributing their quota towards the elucidation of the problem, and the closing years of the century are full of promise and hope for the future. Perhaps the time is not far distant when by intelligent social and legislative measures, by the growth of an enlightened public opinion, and by the spread and diffusion of medical and scientific knowledge, much more may yet be accomplished towards the treatment and prevention of the insanities. Suffice it, however, for the present century that it has completed the humanitarian epoch and inaugurated the scientific. The discoveries of the newest epoch will form the subject of chapters to follow.

